# Role of Pyroptosis in Traumatic Brain and Spinal Cord Injuries

**DOI:** 10.7150/ijbs.45467

**Published:** 2020-04-27

**Authors:** Xinli Hu, Huanwen Chen, Hui Xu, Yaosen Wu, Chenyu Wu, Chang Jia, Yao Li, Sunren Sheng, Cong Xu, Huazi Xu, Wenfei Ni, Kailiang Zhou

**Affiliations:** 1 Department of Orthopaedics, The Second Affiliated Hospital and Yuying Children's Hospital of Wenzhou Medical University, Wenzhou 325027, China; 2 Zhejiang Provincial Key Laboratory of Orthopaedics, Wenzhou 325027, China; 3 University of Maryland School of Medicine, Baltimore, MD 21201, USA; 4 Pediatric Research Institute, The Second Affiliated Hospital and Yuying Children's Hospital of Wenzhou Medical University, Wenzhou 325027, China

**Keywords:** Pyroptosis, Traumatic brain injury, Spinal cord injury, Inflammasomes, Cell death

## Abstract

Central nervous system (CNS) trauma, including traumatic brain injury (TBI) and spinal cord injury (SCI), remains a leading cause for morbidity and mortality worldwide. Past research has shown that cell death plays a critical role in the pathophysiology of CNS injuries. More recently, pyroptosis has been identified as a form of programmed inflammatory cell death, and it is a unique form of cell death in various aspects. Mechanistically, pyroptosis can be categorized into canonical (mediated by caspase-1) and non-canonical (mediated by caspase-4/5/11). In canonical pyroptosis, Nod-like receptors (NLRs) inflammasomes play a critical role, and their activation promotes the maturation and secretion of the inflammatory cytokines interleukin-1β/18 (IL-1β/18), cleavage of gasdermin D (GSDMD), and ultimately pyroptotic cell death. Despite a plethora of new knowledge regarding pyroptosis, detailed understanding of how pyroptosis is involved in CNS injuries and possible ways to improve clinical outcomes following CNS injuries remain elusive. This review discusses the current knowledge on how pyroptosis is involved in CNS injuries, focusing on new discoveries regarding how pyroptosis activation occurs, differences between CNS cell types following injury, time-course of inflammatory responses, and key regulatory steps of pyroptosis. In addition, we highlight various investigational agents that are capable of regulating key steps in pyroptotic cell death, and we discuss how these agents may be used as therapies to improve outcomes following CNS trauma.

## Introduction

Neurotrauma is one of the most serious traumatic injuries and is a common cause of long-term disability and death among young adults. Despite clear clinical need, traumatic brain injury (TBI) and spinal cord injury (SCI) are still without adequate treatments 1. These devastating injuries not only impair physical and psychological health, but also place financial burdens on families and society. In the United States alone, the incidences of TBI and SCI are 333 and 26 per 100 000 patients per year, respectively. Together, TBI and SCI incur an economic burden of approximately 23.7 billion on the American economy ($9.2 billion for TBI, and $14.5 billion for SCI) 1. Clinically, the treatments of CNS trauma mainly include methylprednisolone, surgical decompression, supportive medical care, and rehabilitation. However, patient recovery is limited 2, 3. While many experimental therapies (mainly involving growth factors, biomaterials, and cell transplantation etc.) are being explored in basic and translational research settings, results have been disappointing 4, 5. Thus, new ideas and therapeutic targets are needed for the development of new and effective CNS trauma treatments.

While TBI and SCI share many pathophysiological features, the complex mechanism of CNS injury has been a major obstacle for developing treatments 6. The time course of both TBI and SCI include two overall stages. The first stage features direct brain or spinal cord tissue damage by external force, which causes cell membrane rupture and results in irreversible cell injury and tissue necrosis 7, 8. This is followed by the second stage, during which cell necrosis induces rapid release of intracellular and intra-axonal contents, such as glutamate, ROS, potassium, and cathepsin B. These substances are highly pro-inflammatory, and thus trigger a strong inflammatory response 9. More specifically, pattern recognition receptors (PRRs) are activated by pro-inflammatory substances released from dead cells, thus inducing neuroinflammation 10. Therefore, cell death, and subsequent inflammation, are central biological processes in CNS injury 11. Classically, three different types of cell death have been widely researched: apoptosis 12, autophagic cell death 13, and necrosis 11. These classifications are based largely on morphology, with apoptosis featuring cell shrinkage and chromatin condensation 14, autophagic cell death featuring cytoplasmic vacuolization, phagocytic uptake and lysosomal degradation 15, and necrosis featuring cell swelling, loss of cell membrane integrity, DNA degradation and release of cytoplasmic content 16. More recently, researchers have identified detailed mechanisms regarding programmed necrotic cell death, which is characterized as mitochondrial permeability transition (MPT) dependent necrosis, necroptosis, ferroptosis, and pyroptosis 17. Among these novel mechanisms, accumulating evidence suggests a critical role of pyroptosis, a process of programmed inflammatory cell death, in CNS trauma. In this review, we summarize recent studies of pyroptosis in CNS injuries, which have begun to elucidate how pyroptosis is involved in CNS trauma and how its regulation may be used as potential novel neuroprotective treatments.

## Overview of Pyroptosis

Pyroptosis was first termed in 2002, and it was first observed in macrophages that underwent a unique caspase-1-programmed cell death following exposure to *Salmonella*. Pyroptosis is different from other forms of programmed cell death especially in morphological and biochemical characteristics 18. Morphologically, classical findings include cell swelling induced by incoming water molecules, formation of pores 10-15 nm in diameter on plasma membranes, and release of pro-inflammatory cytokines (IL-1β and IL-18) from the cytoplasm 19. Biochemically, while apoptotic caspases include initiating apoptotic caspases (caspase-2,8,9,10) and execution apoptotic caspases (caspase-3,6,7), pyroptosis caspases include caspases (caspase-1, -4, -5, -11) with both initiator and effector functions 17. Pyroptosis has been extensively observed and well-studied in endothelial cells (EC), smooth muscle cells (SMC), phagocytes, macrophages, neurons and astrocytes, as well as various other cell types 20. In CNS injuries, however, while researchers have reported evidence of pyroptosis in injured cells, the mechanisms of pyroptosis and the consequences of pyroptosis are less clear.

**Figure 1:**
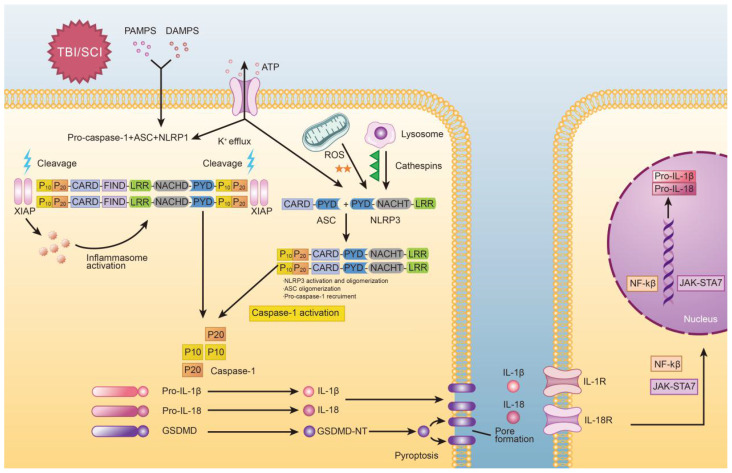
Graphical depiction of the mechanism of pyroptosis in traumatic brain and spinal cord injuries.

The molecular processes of pyroptosis have been described previously and is depicted in Figure 1. Mechanistically, pyroptosis involves two pathways: the canonical caspase-1 inflammasome pathway, and the non-canonical caspase-4/5/11 inflammasome pathway 21. In canonical pyroptosis, which centers around caspase-1, the process is initiated in the “priming step,” where danger or pathogen-associated molecular patterns (DAMPs or PAMPs, respectively) are recognized by Toll-like receptors (TLRs) and Nod-like receptors (NLRs) 22. Once this occurs, expression of inflammasome associated genes is increased, leading to the production of pro-IL-1β and pro-IL-18. Post-translational modifications can also occur during the priming step of canonical pyroptosis 23. After the priming step is complete, the process progresses to the “activation step,” which is centered around inflammasome assembly and caspase activation 24. Here, pro-caspase-1 and adaptor protein apoptosis-associated speck like proteins (ASC) are recruited (often by nod-like receptor family pyrin domain containing 3 (NLRP3)) to form inflammasomes. After that, pro-caspase-1 is cleaved to form caspase-1, which not only promotes cleavage of pro-IL-1β/18 but also cleaves GSDMD into two fragments 25. The N-terminal fragment forms 10-15nm pores in the cell membrane, which eventually leads to discharge of inflammatory factors, cell swelling, membrane rupture 26.

**Figure 2:**
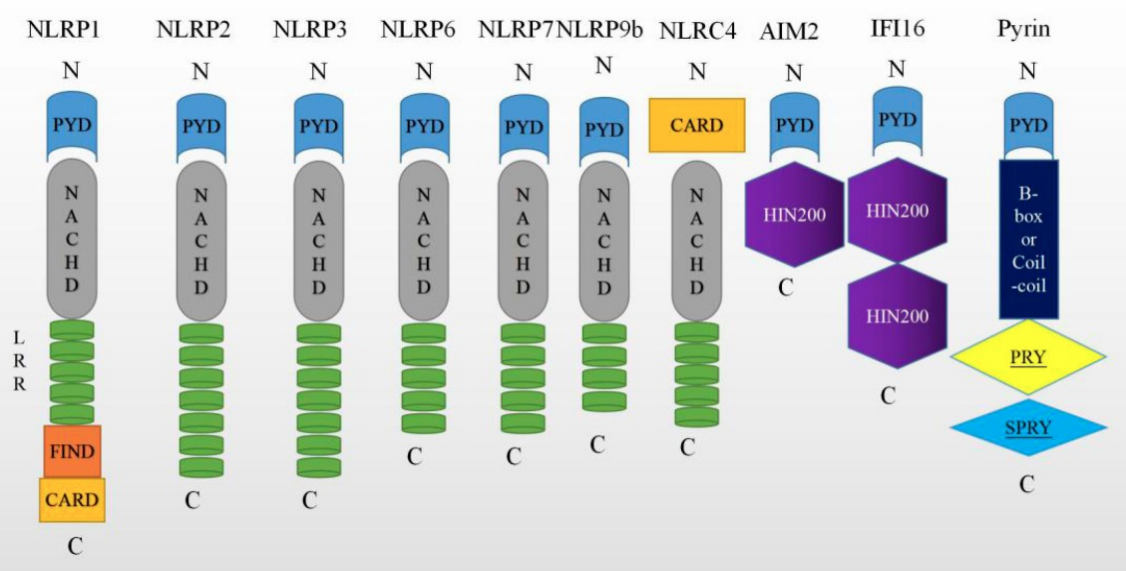
Molecules that can trigger pyroptosis. The nucleotide-binding oligomerization domain (NOD)-like receptors (NLRs) have common NACHT, C-terminal leucine-rich repeat (LRR), and the N-terminal effector domains. It is reported that NLRs have either a pyrin domain or a caspase recruitment domain (CARD). The melanoma 2 (AIM2)-like receptors (AIM2 and IFI16) consist of a C-terminal dsDNA- binding one or two 200 amino acids (HIN200) domain and an N-terminal PYD. Pyrin is a human protein encoded by the MEFV gene, which carries a PYD, 2 B-boxes and a coiled-coil domain, and a SPRY domain.

Overall, caspase-1 can be activated by various inflammasomes including nod-like receptors (NLRs), AIM2-like receptors (ALRs) 27, or tripartite motif family (TRIM). Among these, nucleotide-binding oligomerization domain (NOD)-like receptor (NLR) family have been extensively researched, especially NLRP1 28, NLRP2 10, NLRP3 29, NLRP6 30, NLRC4 31, NLRP9b 32, Pyrin proteins 33 and IFI16 34. These molecules are depicted graphically in Figure 2. While the signaling mechanism may differ slightly depending on the molecules involved, all of these molecules ultimately lead to the activation of caspase-1.

In non-canonical pyroptosis, caspase-4/5 in humans and caspase-11 in mice are involved instead of caspase-1. It is believed that caspase 4/5 and 11 perform similar functions to caspase-1. In the lipopolysaccharide (LPS) induced non-canonical inflammasome pathway, Toll-like receptor 4 (TLR4) recognizes extracellular LPS and caspases recognize cytosolic LPS. Caspase-11/-4/-5 can be directly activated by LPS via binding with their CARD domains, however, it is unclear how LPS is sensed by the CARD domain of caspase-11 35, 36. After the caspases are activated, they then directly cleave GSDMD to initiate pyroptosis 37, 38.

## Activators of Pyroptosis

When external force impacts brain or spinal tissue, immediate compression and contusion occur, accompanied by vascular injury which leads to the destruction of the blood-brain or blood-spinal-cord barrier 39. These events are largely grouped together as “primary injury.” Subsequently, “secondary injury” occurs, which involves the local and systemic release of DAMPs, triggering an inflammatory response via binding to LRR (leucine- rich repeat) domains 40. The mechanism by which NLRP3 inflammasome formation occurs remains unclear. Previous reports have suggested that overproduction of reactive oxygen species (ROS), potassium efflux, and leak of cathepsin are the three main activators of the NLRP3 inflammasome 41. These signaling pathways may not be mutually exclusive, and it is likely that each signal may independently interact with NLRP3 to cause different conformational changes in the molecules involved in inflammasome assembly. Here, we discuss details of how potassium efflux, ROS, and CTSB activate the NLRP3 inflammasome.

### Potassium Efflux

K+ efflux is linked with many known NLRP3 activators including ATP, nigericin and maitotoxin. ATP can bind to the P2X7 receptor, which facilitates an immediate K+ efflux. Subsequently, low intracellular potassium concentration causes changes in NLRP3 inflammasome components, eventually leading to the activation of NLRP3 42-44. Interestingly, NLRP3 is not affected by high extracellular K+ concentrations 45. Importantly, Petrilli* et al* found that inhibiting K+ efflux is an effective way to block NLRP3 inflammasome activation, which may be a strategy that can be used to inhibit pyroptosis in CNS injuries 46. Further work on modulating potassium efflux as a therapeutic approach in CNS injuries is eagerly awaited. NIMA-related kinase 7 (NEK7), a Ser/Thr mitotic kinase, is a modulator which can regulate the activity of NLRP3 inflammasomes and downstream neuroinflammatory responses to K+ efflux. The mechanism of NEK7 involves binding to NLRP3 and then recruiting pro-caspase-1, leading to activation of caspase-1 and inducing pyroptosis. NEK7 may be another mechanism to activate NLRP3 inflammasome 29, 47.

### Reactive Oxygen Species (ROS)

Production of ROS can also trigger NLRP3 inflammasome activation 45. There are many sources of ROS, such as mitochondria, NADPH oxidase, xanthine/xanthine oxidase (X/XO) and incomplete phagocytosis of macrophages. Among these pathways, ROS generated from mitochondria and NADPH oxidase are the most studied 48. Many studies have shown that inhibition of ROS prevents caspase-1 activation and Interleukin 18/1β production. Bae *et al* found that NLRP3 has a disulfide bond that connects the PYD and nucleotide-binding site domains, and is sensitive to altered redox states, suggesting that ROS may trigger NLRP3 inflammasome activation via modifying this disulfide bond 49. Furthermore, evidence suggests that ROS may play a key role in the priming step of pyroptosis and NLRP3 activation 50.

### Release of Cathepsin B (CTSB)

A third trigger for NLRP3 inflammasome activation is the release of CTSB from lysosomes. Jin *et al* demonstrated that lysosomes cannot digest crystals after phagocytosis, and that phagocytosed crystals result in lysosomal swelling and damage, release of CTSB, and activation of NLRP3. Furthermore, this study found that NLRP3 inflammasome activation was not due to the presence of crystals, but rather lysosomal membrane rupture 51. More recently, studies have shown that lack of CTSB markedly reduces NLRP3 activation 52. However, the relationship between CTSB and CNS trauma remains largely unknown. In addition, it was reported that cytoplasmic phospholipase A2 (cPLA2) is activated after SCI and TBI, which can damage lysosome cellular membranes and then lead to leakage of CTSB 53, 54. Thus, it is reasonable to hypothesize that inhibiting CTSB may also curtail pyroptosis in CNS injuries, and that activation of cPLA2 may contribute to CTSB mediated pyroptosis.

## Differential Inflammasome Expression Among Cell Types in CNS Injury

Normal CNS physiology and response to injury involve numerous cell types including neurons, astrocytes, and microglia 55. Following injury, neurons, astrocytes, and microglia generate neurotoxic molecules, such as ROS and CTSB. The formation of NLRP inflammasomes can be activated by these molecules via mechanisms described above. Past studies have shown that there is differential expression of inflammasome components in neurons, astrocytes, and microglia, suggesting that these cells may respond differently to activating signals 56. Xu *et al,* using flow cytometry and immunofluorescence staining, showed that microglia are the main source of NLRP3 inflammasome expression 57, but do not express NLRP1 58. This is in stark contrast to neurons, which mainly express NLRP1-inflammasomes and AIM2, though NLRP3 positive neurons can also be found in rat models of TBI 59. Overall, among known inflammasomes, NLRP1 and NLRP3 are the most commonly studied in TBI and SCI, and they have been shown to play key roles in the innate immune response 60, 61.

Interestingly, astrocytes mainly express the NLRP2 inflammasome, which operates in a unique fashion: ATP binds to P2X7 receptor, opening the pannexin-1 channel, allowing potassium efflux, activating NLRP2, leading to maturation of caspase-1 and secretion of IL-18 and IL-1β. Thus, NLRP2, which is activated by potassium efflux, has a critical role in the astrocyte innate immune response 10. To this end, probenecid, which can inhibit the pannexin-1 channel, has been shown to reduce NLRP2 activation, and may be a potential therapeutic agent for CNS injuries.

## Temporal Pattern of Inflammasome Following CNS Trauma

The formation of inflammasomes and secretion of cytokines following CNS injuries follow a complex temporal pattern. In a moderate parasagittal fluid-percussion injury rat model, maturation of caspase-1, secretion of IL-1β and formation of NLPR1 inflammasome complexes were detected 4 hours following TBI 62. In a TBI rat model, NLRP3 mRNA levels gradually increased following TBI, peaked at 6 hours, gradually decreased until 24 h, and again increased until 7 day after injury; protein levels of NLRP3 were significantly increased from 24 hours to 7 days after injury 59. Finally, in a mouse model of TBI, protein expression of NLRP3, ASC, and caspase-1 were shown to be increased at 1 day following TBI, peaked at 3 days, and gradually decreased over time while levels continued to be elevated compared to control animals 57. Thus, determinants of the temporal pattern of inflammasome formation appears to be multifactorial, and further research is eagerly awaited.

Similarly, temporal patterns of cytokine release following CNS injuries are also highly variable among published results. Overall, TNFα is produced as part of the inflammatory process that occurs early after TBI; TNFα, IL-1β, IL-10 and IL-6 are increased as early as day 1 and decreased at days 2-4 after injury. TGFβ peaks at day 1, and gradually decreases over 21 days. CXCL8 (IL-8) peaks at day 1 and markedly declines at days 2-3, although remaining elevated for up to 108 hours after injury. Many triggers and brakes of inflammation also follow intricate temporal patterns. For example, adenosine increases within hours of injury, but rapidly declines over 12-24 hours. Complement proteins peak at 1 day after injury and decline on days 2-7. Similarly, glutamate peaks at 1 day and declines during days 2-3 63. Data regarding the temporal pattern of inflammasome formation and cytokine release in SCI remain largely unknown.

## Anti-Pyroptotic Therapies

As NLRP3 is believed to be the most commonly involved inflammasome in pyroptosis following CNS injury, development of effective NLRP3 inflammasome inhibitors to treat SCI and TBI has generated significant interest in the scientific community. Various efforts have attempted to make use of the diverse range of candidate targets to inhibit NLRP3 inflammasome activation through the complex signaling pathway of NLRP3 inflammasomes. In particular, therapies may target inflammatory cytokines induced by the NLRP3 inflammasome, GSDMD cleavage to inhibit pore formation, upstream signals, or inflammasome assembly and caspase-1 activation. While there is currently no pyroptosis inhibiting drug approved to treat CNS injuries, many therapies are being developed and hold great promise (Table 1).

### Cytokine Inhibition

As discussed previously, IL-1β signaling is a key step in pyroptosis and is involved in CNS injuries, and thus it has been proposed as a therapeutic target for TBI and SCI. Canakinumab, a monoclonal antibody against IL-1β, inhibits IL-1β binding to the IL-1 receptor, preventing the propagation of downstream inflammatory signals. Past studies have shown that canakinumab can prevent cardiovascular events, however, its possible efficacy in TBI and SCI treatment remains unknown 64, 65. Salidroside, another new experimental agent, has been shown to inhibit pyroptosis via inhibiting inflammatory cytokine expression and NF-κB and MAPK signaling pathways 66.

### GSDMD inhibition

Cleavage of GSDMD after binding to caspase-1 is a critical step in pyroptosis, and it was shown that the cleavage occurs at the FLTD peptide site. Thus, an inhibiting therapy targeting Ac-FLTD-CMK has been designed to block GSDMD cleavage 67. Studies also found that necrosulfonamide (NSA) can interact with cleaved GSDMD, inhibiting oligomerization of p30-GSDMD and preventing the formation of pyroptotic pores and thus pyroptosis cell death 68. The efficacy of these agents in CNS injuries is currently unknown and awaits further investigation.

### Caspase-1 inhibition

Past studies have shown that caspase-1 deficiency reduces neuroinflammation and neuronal damage in the acute phase of TBI and SCI, giving justification to develop caspase-1 inhibitors as therapeutic agents. Pralnacasan (VX-740), and its analog VX-765, are peptidomimetic caspase-1 inhibitors that act via covalent modification of the catalytic site of caspase-1, thereby blocking caspase-1 activation and cleavage of pro-IL-1β and pro-IL-18 69, 70. Parthenolide, an agent that inhibits caspase-1 protease activity and ATPase activity of NLRP3, is another potential therapy 71. Unfortunately, the clinical effectiveness of these agents is not promising due to disappointing pre-clinical data 71, 72.

### NLRP3 inflammasome inhibition

Inhibition of the NLRP3 inflammasome, which is a central molecule in many pyroptosis pathways, has also been explored for therapeutic use 46. MCC950, a diarylsulfonylurea-containing compound, is a potent NLRP3 inhibitor. While studies have shown that MCC950 does not decrease the expression of NLRP3, pro-IL-1β, or pro-caspase-1 in early stages of TBI 73, MCC950 repressed all these molecules 24 hours post-injury, strongly inhibiting caspase-1 activity and IL-1β maturation for up to 72 hours post-TBI 74. These data suggest that MCC950 may not have any effect during the priming stage of pyroptosis, instead targeting the subsequent assembly of NLRP3 inflammasomes. Mechanistically, MCC950 binds directly to the Walker B motif within the NACHT domain of NLRP3, thus blocking ATP hydrolysis and inhibit NLRP3 inflammasome formation 75. Interestingly, MCC950 does not affect TNFα secretion and has no impact on the activation of other inflammasomes such as AIM2, NLRP1, and NLRC4 73, 76. 3,4-methylenedioxy-β-nitrostyrene (MNS), identified by He *et al* via screening a kinase inhibitor library, also inhibits the activation of NLRP3. MNS acts similarly to MCC950 and also suppresses ATPase activity, here via interacting with the NOD and LRR domains, and MNS also leaves AIM2 and NLRC4 inflammasomes unaffected 77. Thus, MNS may be another potential therapeutic agent for CNS injuries awaiting further research. Of note, many other NLRP3 inhibitors are also under investigation including glyburide 78, OLT1177 79 and CY-09 80.

### Suppression of upstream signals

Suppression of upstream activators of inflammasomes, such as K+ efflux, ROS, and CTSB, have also been explored as therapeutic options. Research has shown that preventing K+ efflux leads to a significant decrease in the ability of other inflammasome activators to function 51. P2X7R, an extracellular ATP-gated receptor capable of activating NLPR3-inflammasomes and promoting IL-18 and IL-1β release, has been implicated in CNS injuries by possibly forming homo-trimer ion channels to allow K+ efflux (along with Na+ and Ca2+ influx) and subsequently lead to pyroptosis 81. A438079 is an experimental agent that inhibits P2X7R activity, and Jiang *et al* found that P2X7R inhibition leads to a 50% decrease in caspase-1 activation and promotes functional recovery following SCI in mice 42, 82. Furthermore, A438079 not only decreases the expression of NLRP3 and ASC, it also decreases expression of pro-IL-1β and pro-IL18 by inhibiting the NF-κB pathway 83, 84.

Inhibition of ROS-associated inflammasome activation has also been explored as potential treatments. In TBI, acute cerebral glucose hypometabolism occurs in the brain, and utilization of alternative energy substrates such as ketones is a common response 85. The ketones BHB and acetoacetate are generated in the brain as alternative energy sources to enable neuron survival. Interestingly, BHB has been shown to inhibit Drp1 mitochondrial translocation and promote mitochondrial stability, thus inhibiting ROS-associated NLRP3 inflammasome activation 86.

Finally, CTSB inhibitors have also been studied for their ability to curtail pyroptosis. Recent data suggest that the CTSB inhibitor CA-074Me could inhibit NLRP3 inflammasome activation induced by silica and asbestos 51, 87. However, whether CTSB inhibition is effective in treating CNS injuries is currently unknown. Furthermore, as specific mechanism of how CTSB induces pyroptosis is still unclear, further work is needed to develop effective therapies targeting this pathway.

## Conclusion

The role of pyroptosis is very complicated in CNS injuries, and many unanswered questions remain. In this review, we discussed mechanisms involved in the canonical and non-canonical inflammasome pathways and progression of pyroptosis in TBI and SCI. We also highlighted novel agents that may be able to curtail this form of cell death. While canonical pyroptosis involving NLRP3 is thought to be the predominant pathway in CNS injuries, the roles of many other potential pathways such as other NLR inflammasomes and non-canonical pyroptosis caspases may also be significant. To accurately determine the function of pyroptosis after SCI and TBI, transgenic animals with pyroptosis defects, such as caspase-1^-/-^ mice, should be used in future studies. To date, there are only a few studies that have shed light on the role of non-coding RNAs in pyroptosis of the field. Thus, investigating whether microRNA (miRNA) or long noncoding RNAs (lncRNAs) affect pyroptosis, particularly in TBI and SCI, is of great interest. Overall, while more work is needed to elucidate pyroptosis signaling pathways in CNS injuries, current experimental agents hold great promise, suggesting that novel effective therapies may soon become realities.

## Figures and Tables

**Table 1:** Effects of various therapeutic agents on inflammasome signal pathway after TBI and SCI.

Therapeutic agents	Targets	Potential Mechanism
Canakinumab 64 65	IL-1β	Anti-IL-1β antibody
Ac-FLTD-CMK 66	GSDMD	Inhibits GSDMD cleavage by directly binding to the catalytic region of caspase1, caspase4, caspase 5 and caspase11
VX-740 and VX-765 68, 69 and Parthenolide 70	Caspase-1	Compound act by covalent modification of the catalytic cysteine residue in the active site of caspase-1
MCC950 73/ 3,4-methylenedioxy-β-nitrostyrene (MNS) 76/ glyburide 77/ OLT1177 78/ CY09 79	NLRP3	Blocks the ATPase domain of NLRP3 Inhibits NLRP3 ATPase activity by cysteine modification
A438079 82, 83	P2X7R	Blocks potassium(K+) efflux
BHB 85	Mitochondrial and ROS	Inhibits Drp1 mitochondrial translocation and Prevent ROS generation
CA-074Me 51, 86	Cathepsin B	Inhibits cathepsin B
